# The current consensus on the clinical management of intracranial ependymoma and its distinct molecular variants

**DOI:** 10.1007/s00401-016-1643-0

**Published:** 2016-11-17

**Authors:** Kristian W. Pajtler, Stephen C. Mack, Vijay Ramaswamy, Christian A. Smith, Hendrik Witt, Amy Smith, Jordan R. Hansford, Katja von Hoff, Karen D. Wright, Eugene Hwang, Didier Frappaz, Yonehiro Kanemura, Maura Massimino, Cécile Faure-Conter, Piergiorgio Modena, Uri Tabori, Katherine E. Warren, Eric C. Holland, Koichi Ichimura, Felice Giangaspero, David Castel, Andreas von Deimling, Marcel Kool, Peter B. Dirks, Richard G. Grundy, Nicholas K. Foreman, Amar Gajjar, Andrey Korshunov, Jonathan Finlay, Richard J. Gilbertson, David W. Ellison, Kenneth D. Aldape, Thomas E. Merchant, Eric Bouffet, Stefan M. Pfister, Michael D. Taylor

**Affiliations:** 1 Division of Pediatric Neurooncology, German Cancer Research Center (DKFZ), Heidelberg, Germany; 2 Department of Pediatric Oncology, Hematology and Immunology, University Hospital Heidelberg, Heidelberg, Germany; 3 German Cancer Consortium (DKTK), Heidelberg, Germany; 4 Department of Stem Cell Biology and Regenerative Medicine, Lerner Research Institute, Cleveland Clinic, Cleveland, OH USA; 5 Department of Molecular Medicine, Cleveland Clinic Lerner College of Medicine of Case Western Reserve University, Cleveland, OH USA; 6 Division of Neurosurgery, Arthur & Sonia Labatt Brain Tumour Research Centre, The Hospital for Sick Children, Toronto, ON Canada; 7 Division of Hematology/Oncology, Hospital for Sick Children, Toronto, ON Canada; 8 Arnold Palmer Hospital, Orlando, FL USA; 9 Royal Children’s Hospital, Melbourne, VIC Australia; 10 Department of Pediatric Hematology and Oncology, University Medical Center Hamburg-Eppendorf, Hamburg, Germany; 11 Department of Oncology, St Jude Children’s Research Hospital, Memphis, TN USA; 12 Center for Cancer and Blood Disorders, Children’s National Medical Center, Washington, DC USA; 13 Pediatric Neuro-Oncology Centre Léon Bérard, Lyon, France; 14 Department of Neurosurgery and Institute for Clinical Research, Osaka National Hospital, Osaka, Japan; 15 Fondazione IRCCS-Istituto Nazionale dei Tumori, Milan, Italy; 16 Laboratory of Genetics, Pathology Unit, S. Anna General Hospital, Como, Italy; 17 National Cancer Institute, National Institute of Health, Bethesda, MD USA; 18 Division of Human Biology, Fred Hutchinson Cancer Research Center, Seattle, WA USA; 19 Division of Brain Tumor Translational Research, National Cancer Center Research Institute, Tokyo, Japan; 20 Department of Radiological Sciences, Oncology and Anatomical Pathology, Sapienza University, Rome, Italy; 21 Département de Cancérologie de l’Enfant et de l’Adolescent, Gustave Roussy, Univ. Paris-Sud, Université Paris-Saclay, Villejuif, France; 22 UMR8203 “Vectorologie and Thérapeutiques Anticancéreuses”, CNRS, Gustave Roussy, Univ. Paris-Sud, Université Paris-Saclay, Villejuif, France; 23 Department of Neuropathology, University of Heidelberg, Heidelberg, Germany; 24 Clinical Cooperation Unit Neuropathology, German Cancer Research Center (DKFZ), Heidelberg, Germany; 25 Children’s Brain Tumour Research Centre, The Medical School, University of Nottingham, Nottingham, UK; 26 Department of Pediatrics, University of Colorado Denver, Aurora, CO USA; 27 Nationwide Children’s Hospital and the Ohio State University, Columbus, OH USA; 28 Li Ka Shing Centre, CRUK Cambridge Institute, University of Cambridge, Cambridge, UK; 29 Department of Pathology, St Jude Children’s Research Hospital, Memphis, TN USA; 30 Laboratory Medicine and Pathobiology, University of Toronto, Toronto, ON Canada; 31 Department of Radiological Sciences, St Jude Children’s Research Hospital, Memphis, TN USA

**Keywords:** Ependymoma, Subgroups, RELA, YAP1, Treatment, Trial, Posterior fossa

## Abstract

Multiple independent genomic profiling efforts have recently identified clinically and molecularly distinct subgroups of ependymoma arising from all three anatomic compartments of the central nervous system (supratentorial brain, posterior fossa, and spinal cord). These advances motivated a consensus meeting to discuss: (1) the utility of current histologic grading criteria, (2) the integration of molecular-based stratification schemes in future clinical trials for patients with ependymoma and (3) current therapy in the context of molecular subgroups. Discussion at the meeting generated a series of consensus statements and recommendations from the attendees, which comment on the prognostic evaluation and treatment decisions of patients with intracranial ependymoma (WHO Grade II/III) based on the knowledge of its molecular subgroups. The major consensus among attendees was reached that treatment decisions for ependymoma (outside of clinical trials) should not be based on grading (II vs III). Supratentorial and posterior fossa ependymomas are distinct diseases, although the impact on therapy is still evolving. Molecular subgrouping should be part of all clinical trials henceforth.

## Introduction

**Fig. 1:**
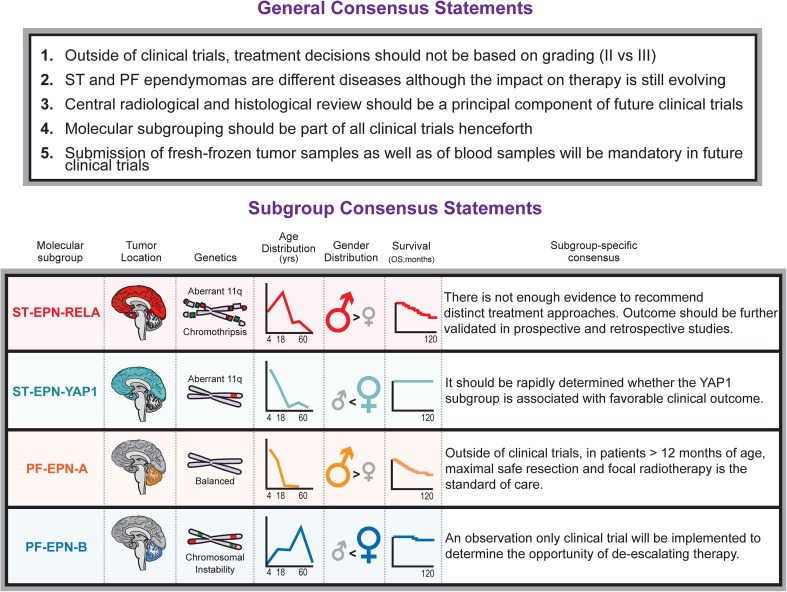
General and molecular subgroup specific consensus statements on the clinical management of intracranial ependymoma

Ependymoma is a histologically defined intrinsic tumor that involves the three major anatomic compartments (supratentorial brain, posterior fossa, and spinal cord) of the central nervous system and affects both children and adults. The current standard of care therapy for patients with intracranial ependymoma remains surgical resection combined with radiotherapy. The survival benefit of chemotherapy for ependymoma and the prognostic ability of histopathological grading criteria to risk-stratify patients are still both inconclusive and contentious. No molecular or tumor-specific immunohistochemical markers are in routine current clinical use for ependymoma. Recent advances in the biological characterization of ependymal tumors have demonstrated the existence of nine clinically, demographically, and molecularly distinct entities, with three occurring in each anatomic compartment. These findings offer new opportunities to create a precise, reliable, and objective platform for stratification of ependymoma patients, and the potential for altering therapeutic decisions based on molecular features. Herein, we discuss the current consensus on the molecular subgroups of intracranial ependymoma (WHO Grade II/III) in children and adults, as well as recommendations for integration into future clinical trial designs. These discussions and recommendations were made by a collection of neuro-oncologists, neurosurgeons, neuro-pathologists, radiation oncologists, and basic scientists, meeting at the global ependymoma consensus conference (Huntsville, Ontario, Canada in September 2015) (Fig. 1).

## The utility of histologic grading of ependymoma in a molecular era

Ependymomas from throughout the central nervous system are currently sub-divided by three histology-based grades used to predict the natural course of the disease and patient outcome [19]. However, the utility of histological grading of ependymoma for risk stratification has been controversial and without consistent associations of tumor grade with patient outcome. The World Health Organization (WHO) Grade I tumors include myxopapillary ependymoma, which typically occurs in the spine, as well as subependymoma, which is usually intracranial. Grade I ependymomas are relatively easier to distinguish, occur predominantly in adults, and are associated with favorable clinical outcomes [19]. Conventional ependymomas are divided between WHO Grade II and WHO Grade III (anaplastic) tumors, the latter showing elevated mitotic activity, microvascular proliferation, and tumor necrosis. Analysis of multiple cohorts of intracranial ependymoma highlights a wide variance in the utility of the Grade II versus Grade III distinction as a robust prognostic marker [9]. Furthermore, the utility of conventional histologic grading may be confounded by the anatomic compartment [29, 37]. These considerations have raised significant questions as to whether the grading criteria should stratify patients into different therapeutic regimens. It was therefore agreed upon that: (1) treatment decisions for ependymoma should not be based on classification and grading that is solely based on histopathological characteristics (especially, the distinction of Grade II versus Grade III tumors) and (2) central and combined histologic–molecular review and classification should be a principal and integral component of any future clinical trial. Indeed, the updated 4th edition of the WHO classification of central nervous system tumors recognizes the supratentorial molecular variant, ST-EPN-RELA (see next section), as a distinct biological and clinical disease entity [20]. Integrated histo-molecular analyses of ependymal tumors from clinically well-annotated prospective international trial cohorts hold promise for inclusion of additional molecular ependymoma ‘entities’ into the upcoming 5th edition of the WHO classification of CNS tumors.

## Molecular subgroups of ependymal tumors in the central nervous system

Although molecular subgroups of ependymoma arising in different anatomical sites exhibit histopathological similarities, their molecular profiles are easily discernable, owing to diverse genetic, transcriptional, and epigenetic programs [7, 8, 18, 22, 24, 30, 36, 37]. Functional cross-species analyses have provided evidence that these molecular differences may be reflective of discrete developmental and cellular origins [16, 30, 33]. Based on demographic, clinical, and molecular data, supported in multiple independent cohorts [23, 29–31, 36, 37], a full consensus was reached that: posterior fossa and supratentorial ependymoma are biologically different diseases both treated by surgery and radiotherapy. Future molecular characterization and clinical trials will assess whether posterior fossa and supratentorial ependymoma may benefit from different forms of therapy. A recent international collaborative study identified nine molecular subgroups of ependymal tumors, three in each anatomical compartment of the central nervous system, spine (SP), posterior fossa (PF), and supratentorial region (ST) [29]. One of the subgroups within each compartment was enriched with WHO Grade I subependymomas (SE), named ST-SE, PF-SE, and SP-SE. These molecular subependymomas occurred in adults only. The two other molecular subgroups within the spine predominantly matched the histopathology-based diagnoses of myxopapillary ependymoma (SP-MPE) and (WHO Grade II/III) ependymoma (SP-EPN). The remaining two molecular types of ependymoma occurred in the posterior fossa, termed PF-EPN-A and PF-EPN-B or alternatively posterior fossa Group A and B, and were independently identified in retrospective studies [36, 37]. PF-EPN-A tumors occur predominantly in infants and young children. Due to their predominant lateral localization, PF-EPN-A tumors are often difficult to completely resect and are associated with high recurrence rates [37]. Conversely, PF-EPN-B tumors occur largely in adolescents and young adults and are associated with a more favorable prognosis. More than 70% of supratentorial ependymomas are characterized by fusions between *C11ORF95* and the *RELA* gene, and were recently termed ST-EPN-RELA [29, 30]. While ST-EPN-RELA tumors may occur in both children and adults, the remaining molecular subgroup of supratentorial ependymoma harbors recurrent fusions to the oncogene *YAP1* and is enriched in the pediatric population [29, 30]. Since preliminary evidence of a small retrospective cohort indicates that patients with YAP1 fusions have an excellent prognosis, it was agreed upon that the international community should move rapidly toward determining whether ST-EPN-YAP1 is a subgroup with an extremely favorable clinical outcome and therefore might benefit from careful therapy de-escalation within the setting of a clinical trial. Retrospective classification of clinically well-annotated supratentorial ependymomas, which have been treated in clinical trials, is expected to give more detailed information on outcome within this subgroup in the near future. No consensus was made upon morphologically diagnosed ST-ependymomas without RELA/YAP1 fusion. It was felt that further investigation was needed for this apparently heterogeneous group of tumors. It was acknowledged that such issues could be addressed with a DNA methylation-based molecular classification for ependymal tumors that represents an unbiased, robust, and uniform scheme that adequately reflects the full biological, clinical, and histopathological heterogeneity across all age groups, grades, and major anatomical CNS compartments. The clinical feasibility of this platform is supported by multiple components: (1) low sample input and DNA requirements, (2) robust results from formalin-fixed paraffin-embedded (FFPE) tissue, and (3) minimal batch effects and assay consistency between different clinical-genomic facilities. In addition to DNA methylation patterns, DNA copy number profiles can be derived from this analysis. It is important to note that chromosome 1q gain has been shown to be an independent prognostic factor that occurs in a subset of PF-EPN-A, PF-EPN-B, and ST-EPN-RELA tumors [12, 17, 24, 29, 32, 37]. Future integrated molecular efforts will explore the integration of molecular subgroup, copy number alterations (namely chromosome 1q gain), and their impact on patient outcome.

Molecular sub-classification is expected to significantly support treatment decisions and simplify risk stratification processes in the immediate future, and should impact clinical trial design and operation in both children and adults. A complete consensus was reached that molecular subgrouping should be a part of all clinical trials moving forward. It was agreed that certification of diagnostic assays for molecular subgroup detection is of high importance. However, it was acknowledged that there were differences between countries regarding certifying agencies and regulations, and therefore most attendees felt that it was not reasonable and feasible to generate a consensus statement on certification processes. To further improve molecular diagnostics and identify new prognostic factors and therapeutic targets, optimal tissue material for ongoing and future biologic discovery studies is required. The great majority of attendees agreed that submitting fresh-frozen samples should be mandatory within upcoming clinical trials for ependymoma. Although DNA methylation profiling can be performed with FFPE-derived tissue, frozen samples would provide optimal material for use in future applications, such as genome sequencing. The interpretation of any tumor sequencing (from a limited gene panel up to whole genome) would dramatically benefit from a matched control to correct for aberrations inherent to the germline. As such, an agreement among most attendees was established that submission of blood samples should also be mandatory for enrollment in a clinical trial. It should be recognized that arguments were made against the mandate of fresh-frozen tissue, owing to the logistical issues of collection, storage, and submission, particularly in small community centers. Additionally, there were ethical concerns regarding the mandated submission of blood. Attendees recognized that efforts would need to be established to create standard operating procedures in smaller centers to enable reliable collection and submission of frozen tissue. Many of those agreeing on a mandate of frozen tissue and blood argued that given the rapid developments in the field of molecular genetics, with the emergence of increasingly powerful analytical devices and computational tools, the time is now to collect tissue specimens in combination with high-quality clinical data. This would enable the use of such advances to improve the care of future ependymoma patients.

## Clinical management of intracranial ependymoma in the context of molecular subgroups

Clinical management of intracranial ependymomas (WHO Grade II/III) is challenging and the optimal treatment strategy is contentious. Intracranial ependymoma, particularly before administration of any therapy, demonstrates predominantly locally invasive growth patterns and has only very low metastatic potential. Surgery plays a primary role for local tumor control and the extent of neurosurgical resection has been the most consistent independent prognostic factor reported in the last decades [5, 6, 34]. The favorable outcome of patients without residual disease and the large difference in event-free and overall survival between patients with complete versus incomplete resection (up to 50% in some series) have led to the concepts of aggressive de-bulking and second-look surgery. Such neurosurgical procedures may be performed immediately following incomplete initial resection or after a short course of chemotherapy and is currently being systematically evaluated in clinical trials. A comprehensive radiological assessment of the residual disease status is expected to give the highest degree of information to base potential secondary neurosurgical intervention decisions. Attendees agreed that central radiological review of pre- and post-surgical imaging should be a principal component of every clinical trial enrolling patients with ependymoma henceforth.

In addition to surgery, post-operative field radiotherapy dosed at 54–59.4 Gy is considered the standard of care for patients with non-disseminated ependymoma to lower the risk of local recurrence [25]. Radiation margins around the target volume have also decreased from 2.0 to 1.0 cm, with no evidence of increased frequency of tumor relapse [25]. Owing to the challenging localization of ependymoma, particularly in the case of laterally located infant posterior fossa tumors, proton therapy has been explored as a radiation modality to spare proximal neurological structures [21]. In the case of recurrent ependymoma, a retrospective analysis demonstrated that the efficacy of re-irradiation, however, was associated with a decline in patient intellectual function [4].

It should be emphasized that all prior studies that evaluated the therapeutic value of neurosurgical interventions and external beam radiation in posterior fossa ependymoma have not accounted for molecular subgroup affiliation and might therefore be confounded by clinical differences in response to therapy between these subgroups. Data from a current retrospective study on four independent non-overlapping cohorts of posterior fossa ependymomas (*n* = 820 cases) found that patients with either PF-EPN-A or PF-EPN-B tumors benefit from gross total resection, with the survival rates being particularly poor for sub-totally resected PF-EPN-A, even in the setting of radiation therapy [31]. Participants at the conference concluded that for PF-EPN-A tumors in patients older than 12 months of age who are treated outside of clinical trials, maximal safe surgical resection and focal radiotherapy should be defined as the standard of care. Owing to the challenging localization of PF-EPN-A tumors, attendees acknowledged that patients would benefit from being treated in specialized centers by experienced neurosurgeons. Since the study strongly demonstrates that a large subset of patients with PF-EPN-B tumors who received a gross total resection did not recur, even in the absence of radiotherapy, it was agreed that a randomized clinical trial for newly diagnosed and gross totally resected PF-EPN-B ependymoma comparing observation versus standard upfront radiation should be considered. Such a trial would test the possibility of therapy to be de-escalated in some patients with PF-EPN-B ependymoma.

Observation for gross totally resected supratentorial ependymomas has also been advocated based on retrospective series that were not molecularly characterized. For example, a retrospective, multicenter study comprising 92 patients (median age was 17.5 years, range 1–83 years) with gross totally resected and non-anaplastic supratentorial ependymal tumors did not find evidence of decreased progression-free or overall survival with the omission of external beam radiation [11]. The 5–10 year Kaplan–Meier estimated overall survival for the overall cohort was 83.2 and 84.1%, respectively. Another retrospective review of only ten patients (median age 5.6 years, range 1.8–15.6 years), which also included ependymomas diagnosed as WHO grade III, found that in some children with completely resected supratentorial ependymoma, surgery alone may be an acceptable treatment option [35]. The outcomes in the aforementioned series differed from the largest cohort published to date comprising 122 supratentorial ependymal tumors that were classified according to their DNA methylation profiles as ST-EPN-RELA, ST-EPN-YAP1 and ST-SE [29]. Tumors harboring *C11ORF95* gene fusions to *RELA* accounted for more than 70% of supratentorial ependymomas (median age 8 years, range 0–69 years) and were associated with a poor prognosis with 5-year progression-free and overall survival of 29 and 75%, respectively. Interestingly, the level of resection did not significantly affect the outcome within the ST-EPN-RELA-positive subgroup in this retrospective analysis in patient samples collected over a long period of time (>20 years). The two remaining supratentorial subgroups, ST-SE and ST-EPN-YAP1, were restricted only to adults (median age 40 years, range 22–76 years) and predominantly to children (median age 1.4 years, range 0–51 years), respectively, with both of these variants showing an excellent prognosis. As the cited studies and other available collections of single cases markedly differ regarding age distribution, therapy modalities and availability of molecular data, variations in outcome cannot be reliably linked to specific treatment approaches or molecular subgroups. It was, therefore, concluded that there was not enough evidence yet to recommend distinct treatment approaches for ST-EPN-RELA ependymoma. Molecular analyses of supratentorial ependymomas from clinically well-annotated international trial cohorts as well as from large retrospective cohorts with long-term follow-up have now been initiated. The authors expect that this approach will help to clarify questions about the clinical outcome of the molecular variants of supratentorial ependymoma and result in explicit therapy recommendations.

In contrast to surgery and radiotherapy, the role of chemotherapy in the management of ependymoma remains unproven despite extensive investigation. Cohorts of pediatric or adult patients in which the role of chemotherapy was retrospectively analyzed either failed to demonstrate a survival advantage or showed substantial variation between individual patients [3, 13, 28]. Two international randomized trials in children are currently comparing post-irradiation chemotherapy to observation only, SIOP Ependymoma II (Europe) and ACNS0831 (USA). In an attempt to delay radiotherapy in very young children, driven by concerns about long-term treatment toxicity, several groups used post-operative chemotherapy approaches in children under 3 years with 42% being the highest rate of 5-year progression-free survival reached to date [14, 15, 40]. In marked contrast, extension of immediate post-operative high-dose conformal radiotherapy to children under the age of 3 years led to 7-year progression-free survival rates of 77%, albeit long-term follow-up for toxic effects on development are still pending [25]. For this reason, radiotherapy deferral strategies that use chemotherapy have been abandoned in most institutions for children >12 months of age. Initial responses to chemotherapy after subtotal resection have been demonstrated [10] and the ependymoma trial ACNS0831 is currently assessing the role of neoadjuvant chemotherapy and second-look surgery, with a combined chemotherapy regimen of vincristine, cisplatin, etoposide, and cyclophosphamide. To date, there is no chemotherapeutic regimen that can routinely be recommended outside the context of a clinical trial. Since the consensus for therapeutic management in the molecularly well-defined PF-EPN-A subgroup does not include any systemic therapy, it will definitely open new avenues for rather rapid implementation of innovative trials for this devastating disease.

## Model development and novel therapeutics

Because of the recognition that ependymal tumors comprise molecularly distinct subtypes, with potentially distinct clinical management, the generation of subgroup-specific pre-clinical models for the development and assessment of novel therapies is required. The identification of candidate cells of origin for ependymoma has permitted the generation of novel mouse models that can be leveraged for novel therapeutic discovery and evaluation [1, 16, 27, 30]. Ephrin receptor B2 (*EPHB2*)-driven ST ependymoma models—also highly expressed in ST-EPN-RELA tumors—have pinpointed 5-fluorouracil treatment as a potential cytotoxic therapy with efficacy in murine models and is currently being evaluated in early phase ependymoma clinical trials [1, 16, 38]. Owing to the clear genetic drivers of ST-EPN-RELA and ST-EPN-YAP1, transcriptionally faithful mouse models are currently generated, which will create similar opportunities to identify druggable targets against these specific subtypes of ependymoma [30]. In parallel, patient-derived xenograft (PDX) models have been established, permitting further therapeutic evaluation of novel drugs and compounds against ependymoma [2, 26, 39]. In the case of PF-EPN-A, the absence of a clear genetic driver has hampered efforts to create genetic mouse models of the disease. Moving forward, it will be important that pre-clinical models are developed in the context of ependymoma subgroups, such that molecular stratification of these tumors is paired with specific therapeutic targets.

## Conclusions

We now recognize that ependymal tumors from different compartments of the central nervous system are biologically distinct and there are phenotypically divergent subgroups within each anatomic compartment. Future clinical trials, the development of pre-clinical model systems, and the identification and testing of subtype-specific therapeutics must accompany molecular classification to be useful to ependymoma patients and to the neuro-oncology community. The differentiation between histologically defined grade II versus grade III/anaplastic ependymomas is problematic and of limited utility for clinical decision-making, and therefore should be used with great caution outside the setting of a clinical trial. For patients with PF-EPN-A ependymoma over the age of 12 months of age, the recommended standard of care is maximal safe micro-neurosurgical removal followed by local radiotherapy, but probably does not include the routine use of chemotherapy outside the setting of a clinical trial. A subset of PF-EPN-B ependymoma patients who undergo gross total micro-neurosurgical resection are likely cured in the absence of radiotherapy, and a clinical trial to test the possibility to avoid radiotherapy in the context of complete resection for PF-EPN-B patients is indicated. The characteristics and heterogeneity between molecular subgroups of supratentorial ependymoma require additional study before specific treatment recommendations can be made. The division of an already uncommon entity (“ependymoma”) into nine new entities will necessitate great co-operation and international collaboration with the pediatric and adult neuro-oncology community if clinical trials are to be properly and expeditiously completed.
